# Transformation of Follicular Lymphoma to Double Hit B-Cell Lymphoma Causing Hypercalcemia in a 69-Year-Old Female: A Case Report and Review of the Literature

**DOI:** 10.1155/2014/619760

**Published:** 2014-08-04

**Authors:** Sakshi Kapur, Miles B. Levin

**Affiliations:** ^1^Department of Internal Medicine, Overlook Medical Center, 99 Beauvoir Avenue, Summit, NJ 07902, USA; ^2^Division of Pathology, Overlook Medical Center, 99 Beauvoir Avenue, Summit, NJ 07902, USA

## Abstract

Double hit B-cell lymphomas are rare tumors that are defined by a chromosomal breakpoint affecting the MYC/8q24 locus in combination with another recurrent breakpoint, mainly a t(14;18)(q32;q21) involving BCL2. These tumors mostly occur in adults and carry a very poor prognosis. Double hit lymphomas can occur de novo, or arise from transformation of follicular lymphoma. We report a case of a 69-year-old female with abdominal distention and progressively worsening weakness over six months. Patient presented with severe hypercalcemia and multiple intra-abdominal/pelvic masses. Histopathology results of the abdominal mass were compatible with a double hit B-cell lymphoma. However, bone marrow biopsy results showed a low grade follicular lymphoma, thus suggesting peripheral transformation of follicular lymphoma to double hit B-cell lymphoma. Patient was transferred to a tertiary care center and was started on combination chemotherapy (EPOCH: doxorubicin, etoposide, vincristine, cyclophosphamide, and prednisone). Our paper highlights not only transformation of follicular lymphoma to double hit B-cell lymphoma and the challenges encountered in diagnosing and treating these aggressive tumors, but also the association of new onset/worsening hypercalcemia in such patients.

## 1. Introduction

Double hit B-cell lymphomas are rare tumors that are defined by a chromosomal breakpoint affecting the MYC/8q24 locus in combination with another recurrent breakpoint, mainly a t(14;18)(q32;q21) involving BCL2. The partner of BCL2/18q21 breakpoint mostly is the IGH locus at 14q32, and in some cases a t(8;14;18) may be present. Double hit B-cell lymphomas can arise either de novo or from transformation of follicular lymphoma. More than half of the patients present with widespread, often extranodal disease. Patients usually present with poor prognostic factors such as elevated LDH, bone marrow/CNS involvement, and a high international prognostic index score. Double hit B-cell lymphomas usually show a poor response to standard chemotherapy regimens, otherwise used for treating B-cell lymphomas.

## 2. Case Report

A 69-year-old Caucasian female presented to our hospital with complaints of progressively worsening abdominal distention over one month. Patient also complained of worsening confusion and weakness over the last few days. Review of systems was positive for loss of appetite and 20 IB weight loss over the last six months. Physical examination revealed an average sized female with no acute distress. Vital signs were as follows: temp.: 98.4 F, pulse: 110 beats per minute, blood pressure: 110/74 mm of Hg, and a respiratory rate of 16 per minute. Head and neck exam revealed dry mucous membranes; however no thyromegaly or lymph node enlargement was noted. The abdomen appeared to be distended on exam, and faint bowel sounds were present in all the four quadrants. Multiple masses were palpated through the anterior abdominal wall; however no guarding or rigidity was noted. The abdominal exam was also negative for hepatosplenomegaly. Both heart and lungs were normal on exam. Cranial nerves 2–12 were intact and no focal deficits were noted.

Laboratory work-up was as shown in Table 1.

**Figure 1:**
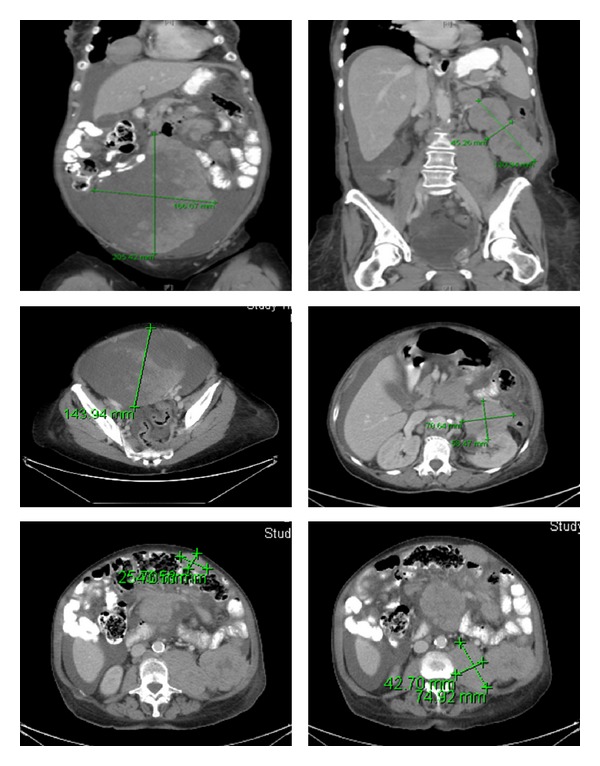
Computer tomography of the abdomen showing multiple intra-abdominal/pelvic masses and massive ascites.

Computer tomography of the abdomen (CT) showed severe degree of ascites, a large heterogeneous intra-abdominal/pelvic mass extending into the lower abdomen measuring 20 cm in craniocaudal dimension, 17 cm in transverse width and 14 cm in anteroposterior depth, and multiple intra-abdominal cystic/pelvic masses representing intraperitoneal implants. Along the left anterior abdominal wall, a 6.5 cm × 4.5 cm mass was seen invading the left kidney and extending caudally. Bilateral pleural effusions were also seen (Figure 1).

Patient was started on intravenous fluids, diuretics, calcitonin, and pamidronic acid. An oncology consultation was obtained and further work-up revealed normal urine analysis, normal urine and serum protein electrophoresis, LDH: 1593 U/L (normal range: 100–190), PTH (intact): <2.5 pg/mL (normal range: 14–72), and serum ferritin of 391 *μ*g/L (normal range: 3–105 *μ*g/dL). Tumor markers were as follows: Ca-125 370.9 U/mL (normal range: 0–30.2), CEA < 2.0 *μ*g/L (normal range: 0–3), and Ca 19-9 5.1 U/mL (normal range: 0–35).

**Figure 2:**
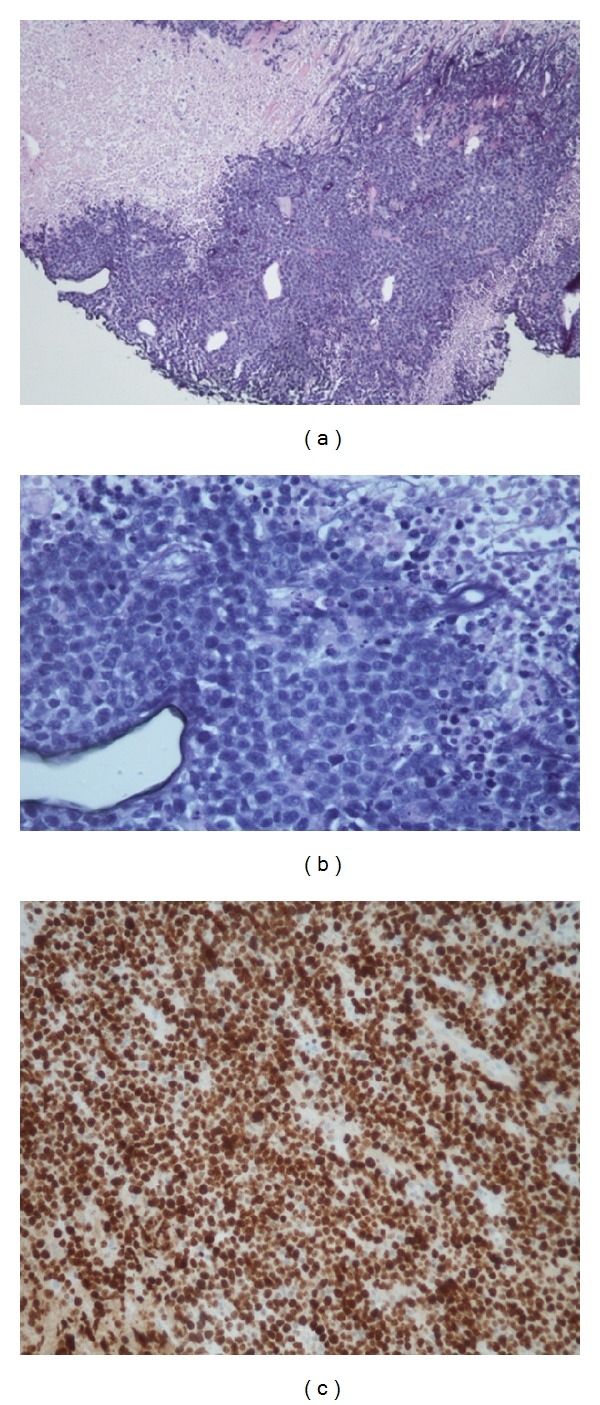
(a) 100x H&E image shows a monomorphic infiltrate of small round blue cells with adjacent necrosis, (b) 400x H&E high power demonstrates medium sized cells with admixed apoptotic bodies and mitotic figures reminiscent of Burkitt or a Burkitt-like lymphoma, and (c) Ki-67: high proliferation rate over 90% supports the morphologic impression and is indicative of an aggressive lymphoma.

**Figure 3:**
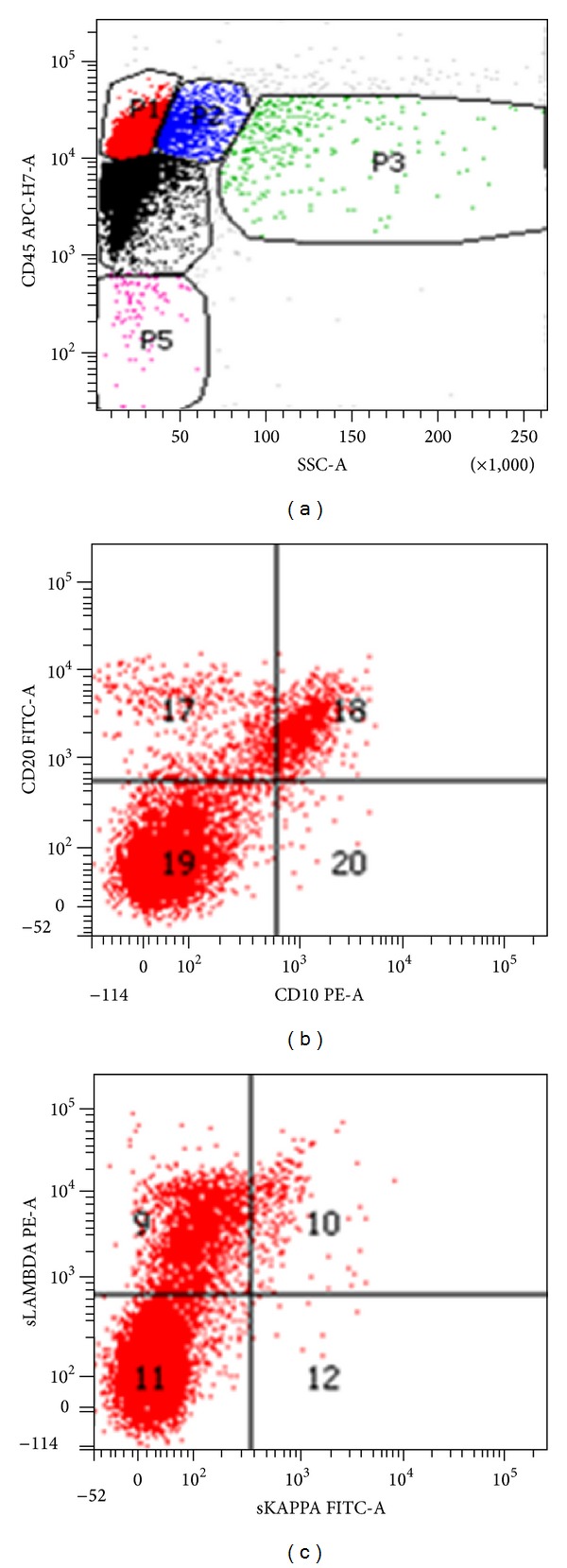
(a) Flow cytometry of the ungated specimen of the abdominal mass showing a Cd45 bright population, compatible with a lymphoproliferative process (rather than a carcinoma, melanoma, etc.), (b) gating only the CD45 bright/low side scatter population shows a predominant CD20+/CD10+ population, consistent with a B-cell population of germinal center origin, and (c) light chain expression shows lambda light chain restriction, diagnostic of a B-cell lymphoma.

**Figure 4:**
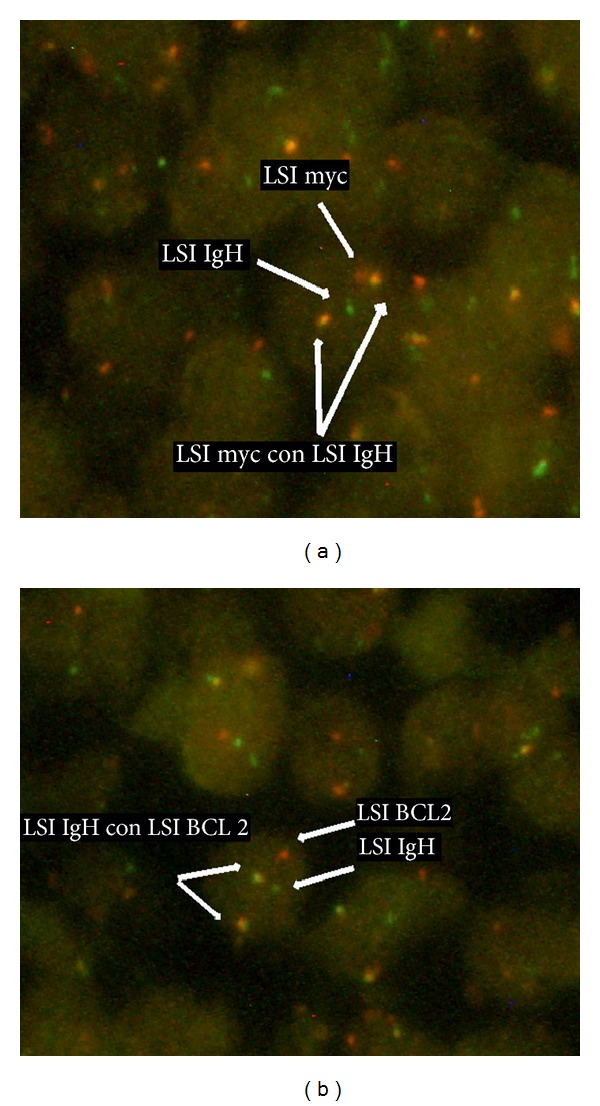
FISH analysis using Abbott Tri-color Dual fusion Translocation Probe hybridized to a nucleus revealing, (a) an* IgH/c-*myc rearrangement by the juxtaposition of the red (c-myc) and green (IgH) yielding the yellow fusion. One native c-myc (red) and native IgH (green) are also present; (b) a* BCL-2*/*IgH* rearrangement by the juxtaposition of the red (*BCL-2*) and green (*IgH*) yielding the yellow fusion. One native* BCL-2* (red) and native* IgH* (green) are also present.

A CT guided core needle biopsy of the abdominal mass was performed. Histopathology results showed a dense lymphoid infiltrate comprising of medium-sized cells with increased mitotic figures and apoptotic bodies with adjacent geographic necrosis. Immunohistochemical stains on neoplastic cells were positive for CD20, CD45, CD10, BCL2, BCL6, and PAX8 and negative for cyclinD1, CD5, and weakly positive expression for MUM1. The ki-67 proliferation rate was 95% (Figure 2), findings consistent with a high grade lymphoma such as Burkitt's or a B-cell lymphoma, unclassifiable with features intermediate between diffuse large diffuse B-cell lymphoma and Burkitt lymphoma. Flow cytometry of the abdominal mass was also suggestive of an aggressive B-cell lymphoma (Figure 3). Subsequent FISH testing was positive for t(8;14), c-myc-IgH translocation, and a t(14;18) IgH–BCL2 translocation (Figure 4). These findings were diagnostic of the so-called “*Double hit B-cell lymphoma,*” WHO recognized category of B-cell lymphoma, unclassifiable with features intermediate between diffuse large B-cell lymphoma and Burkitt lymphoma.

**Figure 5:**
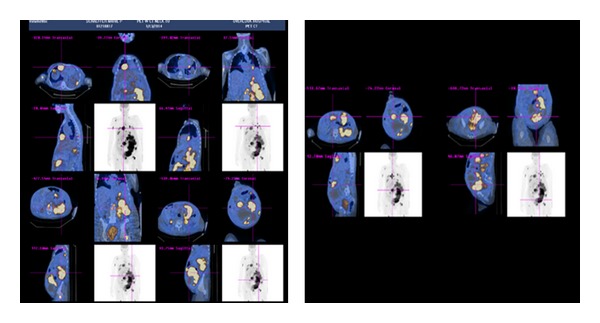
WHOLE BODY PET-CT showing multiple areas of hypermetabolic activity in the abdomen with hypermetabolic bony lesions suspicious of extensive metastatic disease.

A whole body PET-CT revealed large hypermetabolic confluent nodal masses involving the left perirenal and peripancreatic regions with extension into the abdominal mesentery, a very large hypermetabolic mass in the anterior abdominopelvic midline extending into the left anterior abdominal wall, hypermetabolic soft tissue masses implanted along the diaphragm bilaterally, and focal hypermetabolic activity within the lateral wall of the left ventricle of the heart. Multiple foci of hypermetabolic activity throughout the bones, suspicious for osseous metastatic disease, were also noted (Figure 5).

**Figure 6:**
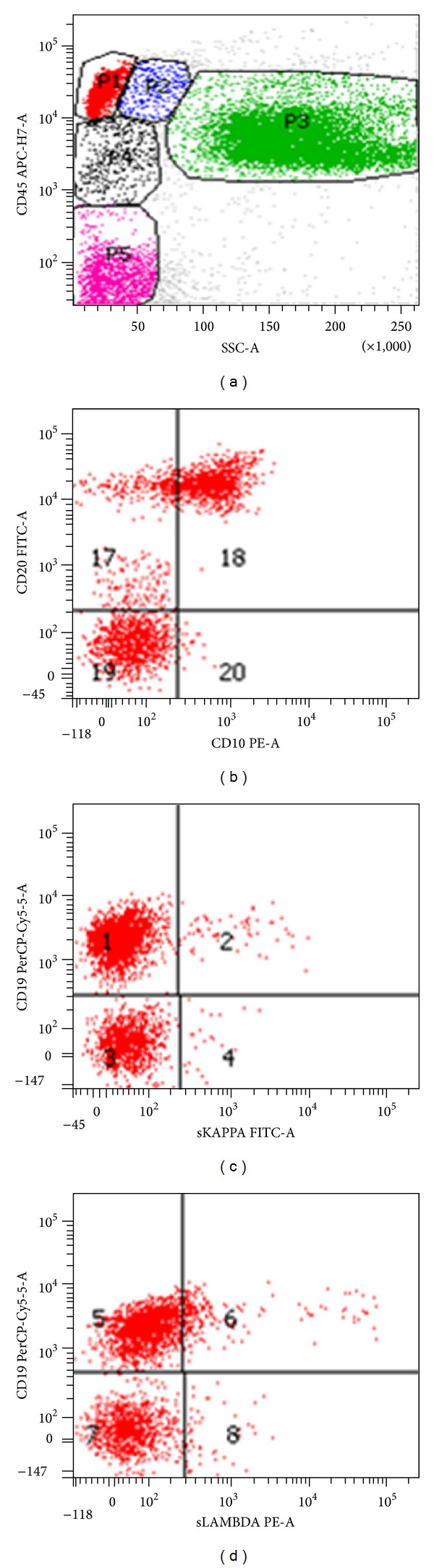
(a) Flow cytometry of the ungated bone marrow aspirate shows 16% cellularity in the CD45 bright/low side scatter gate, (b) gating only the CD45 bright/low side scatter gate shows a population of CD10+/CD20+ lymphocytes representing 7% of overall cellularity, and (c) this population shows dim surface lambda light chain restriction, all findings consistent with a low grade lymphoma of germinal center origin.

However, bone marrow biopsy and aspirate showed a sprinkling of small B-cells comprising 10% of marrow cellularity without significant aggregate formation. The corresponding flow cytometry (Figure 6) demonstrated clonality with a lambda restricted CD20+/CD10+ population consistent with a low grade B-cell lymphoma with a germinal center phenotype. Thus, these findings were most consistent with an underlying follicular lymphoma, with subsequent peripheral transformation into an aggressive nodal-based “double hit” lymphoma.

Following hydration, diuretics, calcitonin, and pamidronic acid, patient's serum calcium started trending down, and her kidney function showed improvement.

As per recommendation by oncology, patient was transferred to a tertiary care center. She was started on combination chemotherapy comprising of EPOCH (doxorubicin 10 mg/m^2^ *plus* etoposide 50 mg/m^2^ *plus* vincristine 0.4 mg/m^2^ by continuous IV infusion on days 2–4* plus* cyclophosphamide 750 mg/m^2^ on day 6* plus* prednisone 60 mg/m^2^ on days 1–6, every 21 d). Patient also received intrathecal methotrexate for CNS prophylaxis.

## 3. Discussion

Double hit lymphomas (DHLs) are defined by a chromosomal breakpoint affecting the MYC/8q24 locus in combination with another recurrent breakpoint, mainly a t(14;18)(q32;q21) involving BCL2. The partner of BCL2/18q21 breakpoint mostly is the IGH locus at 14q32, and in some cases a t(8;14;18) may be present [1–4]. Many DHLs arise in patients with prior follicular lymphoma often with known BCL2 translocations, though de novo lymphomas are also known to occur [5, 6]. Lymphomas with double hit genotype include Burkitt or Burkitt-like lymphoma, diffuse large B-cell lymphoblastic lymphoma, TdT+ B-cell lymphoblastic lymphoma, low grade follicular lymphoma, and plasmablastic lymphoma [1, 2]. DHLs are relatively infrequent and mainly occur in adults.

More than half of the patients present with widespread, often extranodal disease. Unlike Burkitt lymphoma, there is no preferential localization in the ileocecal region or jaws. Magro et al. reported three patients with an aggressive form of B-cell lymphoma secondarily involving the skin. Two patients had an antecedent and/or concurrent history of follicular lymphoma, and one patient developed a de novo lymphoma. In each case there was a c-MYC and BCL2/IGH rearrangement, diagnostic of DHL [7]. Kaplan et al. reported another case of a 53-year-old male who presented with abdominal pain, shortness of breath, night sweats, ascites, and extensive lymphadenopathy. Cytologic examination of the peritoneal fluid showed two distinct populations of neoplastic cells, findings compatible with a B-cell double hit lymphoblastic lymphoma [8].

Patients usually present with lymphadenopathy and/or mass lesions in extranodal sites. Some patients may have a leukemic presentation. DHLs show frequent involvement of the bone marrow, peripheral blood, and CNS and are usually associated with a very poor prognosis. Patients present with poor prognostic factors such as elevated LDH, bone marrow/CNS involvement, and a high international prognostic index score [9]. Most studies on larger series of patients suggest a poor prognosis, also if treated with R-CHOP (rituximab, cyclophosphamide, doxorubicin, vincristine, and prednisolone) or high intensity treatment modalities [10].

Transformation of follicular lymphoma to a more aggressive non-Hodgkin lymphoma is a pivotal event in the natural history of follicular lymphoma [11–16]. Follicular lymphomas are mainly known to transform to diffuse large B-cell lymphoma, to B-cell unclassifiable lymphoma (DHLs), and very rarely to lymphoblastic lymphoma and acute lymphoblastic leukemia. Al-Tourah et al. established a clinical diagnosis of transformation based on the presence of at least one of the following: sudden rise in LDH, rapid discordant localized nodal growth, new involvement of unusual extranodal sites, new B symptoms, and new hypercalcemia [17]. However, in some patients these symptoms may occur with progression of follicular lymphoma and not necessarily with transformation. Several studies have reported higher risk of transformation in patients with advanced disease stage, presence of B symptoms and bulky disease, high *β*2 microglobulin and low albumin levels, and higher scores of follicular lymphoma international prognostic index [18, 19]. Also, early initiation of follicular lymphoma treatment does not decrease the risk of transformation. The median time from diagnosis to transformation in the reported series ranges from 40 to 66 months [16].

The optimal treatment for these lymphomas remains undefined. DHL responds poorly to R-CHOP regimens, CODOX-M/IVAC regimens, and Hyper-CVAD chemotherapy [20]. Most patients following transformation are treated with standard doxorubicin containing combination chemotherapy regimens and a complete remission up to 40% has been reported [21]. Radiation, either alone or in combination with chemotherapy, has also been used in patients with limited disease, and a complete remission rate of 70% has been reported [21]. Radioimmunotherapy using radioactive nucleotide labeled antibodies such as yttrium Y^90^ ibritumomab and iodine I^131^ tositumomab has shown to exhibit antilymphoma activity in a small number of patients with transformed follicular lymphoma [22]. High dose chemotherapy and autologous stem cell transplantation have also been evaluated for the treatment of patients with transformation of follicular lymphoma [23]. Parker et al. reported two cases of DHL successfully treated with aggressive immunochemotherapy followed by autologous stem cell transplantation and radiation therapy [24]. However, more research is needed before these treatment modalities can be widely accepted to treat patients with these rare lymphomas.

Hypercalcemia of malignancy occurs in 20–30% of cancer patients [25]. It occurs in patients with solid tumors as well as hematological malignancies. Various mechanisms are known to cause hypercalcemia in malignancy; these include osteolytic metastasis, tumor secretion of parathyroid hormone related protein (PTHrP), and tumor production of 1, 25-dihydroxyvitamin D (calcitriol). The most common cause of hypercalcemia in patients with nonmetastatic solid tumors and in some patients with non-Hodgkin lymphoma is secretion of PTHrP, a condition called humoral hypercalcemia of malignancy [26]. However, excessive production of calcitriol is the most common cause of hypercalcemia in patients with Hodgkin lymphoma and approximately one-third of patients with non-Hodgkin lymphoma [27]. New onset/worsening hypercalcemia is a very rare manifestation of follicular lymphoma transformation to double hit B-cell lymphoma [17].

Our paper highlights not only peripheral transformation of follicular lymphoma to B-cell DHL and the challenges encountered in diagnosing and treating these aggressive tumors, but also the association of new onset/worsening hypercalcemia in such patients.

## 4. Conclusion

Double hit B-cell lymphomas are rare tumors, which can arise either de novo or following transformation of follicular lymphoma. These lymphomas usually occur in adults and often carry a very poor prognosis. Our paper highlights a case of a 69-year-old female who presented with abdominal distention and weakness over six months. Although biopsy of the abdominal mass revealed findings compatible with a double hit lymphoma, bone marrow biopsy results showed a low grade follicular lymphoma, thus suggesting peripheral transformation of follicular lymphoma to double hit B-cell lymphoma.

## Figures and Tables

**Table 1:** Laboratory work-up.

Hb/Hct	9.5 gm/dL/30.1% ↓
WBC	6.34/nL N
Platelet count	321/nL N
BUN	21 mg/dL ↑
Creatinine	1.4 mg/dL ↑
Calcium	16.3 mg/dL ↑
Ionized calcium	7.87 mg/dL ↑
Phosphorus	2.6 mg/dL ↓
Magnesium	1.5 mg/dL ↓
Potassium	3.6 mmol/L N
Sodium	140 mmol/L N
Uric acid	9.1 mg/dL ↑
Glomerular filtration rate	40 mL/min/1.73 m^2^ ↓
Albumin	3.8 gm/dL ↓

N: normal.
